# NURSE-AI: A Nurse-by-Design Framework for Multi-Sensor, AI-Enabled Chronic Wound Assessment in Community Healthcare

**DOI:** 10.3390/s26102948

**Published:** 2026-05-08

**Authors:** Chiara Barchielli, Sara Jayousi, Riccardo Mari, Beatrice Albanesi, Marco Alaimo, Gianluca Galeotti, Paolo Zoppi, Lorenzo Mucchi

**Affiliations:** 1 Management and Health Laboratory, Institute of Management, Sant’Anna School of Advanced Studies of Pisa, 56127 Tuscany, Italy; 2 PIN Foundation, Prato Campus of University of Florence, 59100 Prato, Italy; sara.jayousi@pin.unifi.it; 3 Azienda USL Toscana Centro, 50122 Florence, Italy; riccardo.mari@uslcentro.toscana.it (R.M.); marco.alaimo@uslcentro.toscana.it (M.A.); gianluca.galeotti@uslcentro.toscana.it (G.G.); paolo.zoppi@uslcentro.toscana.it (P.Z.); 4 Department of Public Health and Pediatrics, University of Turin, 10124 Turin, Italy; beatrice.albanesi@unito.it; 5 Department of Information Engineering, University of Florence, 50139 Florence, Italy; lorenzo.mucchi@unifi.it

**Keywords:** multi-sensor imaging, RGB–D, LiDAR, thermal imaging, hyperspectral imaging, artificial intelligence, chronic wounds, community healthcare, nursing workflow, wound bed preparation, synthetic data, interoperability, MDR, GDPR

## Abstract

**Highlights:**

**What are the main findings?**

- This study proposes NURSE-AI, a Nurse-by-Design methodological framework for evaluating AI-enabled multi-sensor wound assessment systems in community healthcare.
- Feasibility evaluation using synthetic multimodal scenarios demonstrated robust segmentation performance, reliable wound measurement, and good usability among community nurses.

**What are the implications of the main findings?**

- The framework provides a structured pathway linking sensor technologies, AI interpretability, and nursing workflow integration for community-based wound assessment.
- This approach may facilitate future clinical validation and scalable deployment of AI-assisted wound assessment systems in community healthcare.

**Abstract:**

Accurate and reproducible chronic wound assessment remains challenging in community healthcare, where environmental variability and subjective visual evaluation may introduce substantial measurement errors. Although multi-sensor technologies, including RGB–D imaging, mobile Light Detection and Ranging (LiDAR), thermal infrared imaging, and hyperspectral sensing, as well as artificial intelligence (AI)-based analytics, have advanced considerably, real-world adoption remains limited because of workflow misalignment, insufficient interpretability, and regulatory complexity. This study presents NURSE-AI, a Nurse-by-Design methodological framework for evaluating and preparing multi-sensor, AI-enabled wound assessment systems for deployment in community healthcare. NURSE-AI is proposed as a pre-implementation methodological framework supported by a feasibility study based on a synthetic dataset; therefore, it is not a clinical validation study, and no patient data were used. The framework integrates: (i) a GDPR-compliant synthetic multimodal dataset including RGB, depth, thermal, and hyperspectral-proxy layers; (ii) workflow-embedded acquisition modeling tailored to Family and Community Nurses (FCNs); (iii) a Wound Bed Preparation (WBP)-aligned interpretability layer; and (iv) a governance-by-design checklist addressing interoperability, metadata traceability, and regulatory readiness under Regulation (EU) 2017/745. A mixed-method feasibility evaluation was conducted with community nurses within AUSL Toscana Centro (Italy). The System Usability Scale (SUS) yielded a mean score of 74.5 ± 6.2, indicating good usability. Synthetic multimodal evaluation demonstrated promising segmentation performance under controlled synthetic conditions, with Intersection over Union (IoU) values ranging from 0.87 to 0.93, and simulated Intraclass Correlation Coefficient (ICC) values ≥ 0.90 for wound area estimation. Agreement between AI-generated WBP mappings and nurse interpretation ranged from κ = 0.80 to κ = 0.84. The NURSE-AI framework proposes a structured and reproducible pathway connecting sensor innovation, AI interpretability, nursing workflow integration, and regulatory preparedness, thereby providing structured groundwork for future clinical validation and scalable deployment in community healthcare.

## 1. Introduction

### 1.1. Context

Chronic wounds represent a major clinical and socioeconomic burden because they are associated with prolonged healing times, increased risk of complications, reduced quality of life, and substantial healthcare costs [1,2]. In routine care, wound assessment often relies on subjective visual inspection and manual measurements, which may result in inter-rater variability and limited reproducibility. These limitations become even more relevant in community-based care, where clinical evaluations are frequently performed in patients’ homes rather than in controlled healthcare environments. Home-care settings introduce additional sources of variability, including inconsistent lighting, limited physical space, variable patient positioning, and time constraints during home visits. These contextual factors may affect image quality, measurement reliability, and the overall feasibility of standardized wound documentation. For this reason, chronic wound assessment in community healthcare remains a challenging field in which technical innovation must be aligned with everyday nursing practice. Recent advances in multi-sensor imaging technologies have created new opportunities for more objective wound assessment. RGB imaging provides visual and structural information, depth sensing supports geometric reconstruction, thermal imaging may reveal inflammatory patterns, and hyperspectral sensing can offer indirect information about tissue oxygenation and perfusion. In parallel, AI, particularly deep learning, has enabled automated segmentation, classification, and measurement of wounds through data-driven architectures widely adopted in biomedical imaging [3,4,5,6,7,8,9,10,11]. The recent literature has further shown that deep learning approaches can substantially improve feature extraction, pattern recognition, and quantitative analysis across heterogeneous biomedical datasets, thereby strengthening their relevance for automated wound assessment systems [7,8,9,10,11]. Recent studies have shown that advanced deep learning architectures, including graph-based and information-theoretic approaches, can improve feature extraction and pattern recognition across biomedical datasets [7,8,9,12,13]. These advances support the development of more robust and reliable automated wound assessment systems.

However, despite these promising developments, many experimental systems remain difficult to integrate into real-world community nursing workflows.

### 1.2. Background and Rationale

#### 1.2.1. Implementation Gap Between Algorithmic Performance and Community Deployment

A well-recognized gap exists between the algorithmic performance reported in experimental studies and the practical implementation of wound assessment technologies in community healthcare.

Many wound imaging studies describe high segmentation accuracy or precise geometric reconstruction under controlled conditions, often using standardized lighting, fixed acquisition setups, or robotic scanning systems [3,4,5]. While these studies demonstrate technical feasibility, their operational assumptions often do not reflect the constraints typically encountered in home-care settings.

Community nursing practice is characterized by contextual challenges such as variable lighting conditions, limited physical space, patient mobility or positioning difficulties, short visit duration, and heterogeneous device availability. Consequently, solutions optimized primarily for algorithmic performance may fail to achieve adoption in routine clinical practice if they are not designed with workflow compatibility in mind. Addressing this translational gap requires methodological frameworks that integrate sensor technologies, AI, clinical reasoning, and workflow considerations from the earliest design stages. In response to this need, we introduce NURSE-AI, a Nurse-by-Design framework in which nursing workflow constraints are treated as a primary design driver rather than a secondary implementation issue.

#### 1.2.2. Related Works

##### Automated Wound Measurement

Automated wound measurement using computer vision has received increasing attention in recent years. Smartphone-based imaging pipelines have demonstrated the feasibility of estimating wound area from RGB images, although their accuracy remains sensitive to capture conditions and camera orientation [5]. RGB–D systems and robotic scanning solutions have shown improved geometric accuracy, enabling volumetric wound reconstruction and perimeter measurement [3,4]. However, these systems often depend on specialized hardware configurations and acquisition procedures that may not be practical in home-care environments. Recent advances in deep learning and biomedical image analysis have further strengthened the development of automated segmentation and measurement approaches, with increasing emphasis on robustness, generalization, and applicability in real-world healthcare settings [7,8,9,10,11].

##### Thermal Imaging and Diabetic Foot Monitoring

Thermography has been widely investigated as a tool for detecting inflammatory changes associated with diabetic foot complications. Deep learning models applied to thermal imaging datasets have shown promising classification performance for identifying thermographic patterns associated with ulcer risk [6]. Nevertheless, thermal imaging is rarely integrated into a broader multimodal wound assessment pipeline explicitly aligned with clinical reasoning frameworks used by nurses.

##### Explainability and Clinical Alignment

Explainable AI has been proposed as an essential requirement for improving transparency, trust, and adoption of machine learning systems in healthcare. In clinical settings, algorithmic outputs should not only be accurate but also understandable and meaningful to healthcare professionals. Therefore, aligning AI-derived information with established clinical reasoning models is particularly important for supporting decision-making in wound care [14].

##### Wound Bed Preparation as a Clinical Framework

The WBP model provides a structured framework for chronic wound management, including tissue management, infection or inflammation control, moisture balance, and wound edge advancement [1]. Because it is already familiar to clinicians and nurses involved in wound care, WBP offers a clinically grounded scaffold for mapping sensor-derived signals and AI outputs into actionable assessment domains.

### 1.3. Our Contribution

This study addresses the translational gap between experimental multi-sensor wound imaging systems and their practical adoption in community healthcare. The main contributions of this work are fivefold.

1. First, we propose NURSE-AI, a Nurse-by-Design methodological framework for evaluating multi-sensor wound assessment technologies in community care settings. The framework explicitly incorporates real-world acquisition constraints typical of home environments and integrates workflow guidance tailored to FCNs.
2. Second, we introduce a GDPR-compliant synthetic multimodal dataset generation strategy designed for preclinical evaluation of AI-based wound assessment systems without using patient data. The dataset provides spatially aligned RGB, depth, thermal, and hyperspectral-proxy layers, together with segmentation ground truth and WBP labels, thus enabling controlled testing of segmentation accuracy and measurement reliability.
3. Third, we develop a clinically grounded interpretability layer aligned with the Wound Bed Preparation model, translating sensor-derived signals and AI outputs into clinically meaningful domains for nursing assessment, including tissue status, infection or inflammation indicators, moisture balance, and wound edge progression. This approach aims to improve the transparency and clinical usability of AI-assisted wound assessment.
4. Fourth, we define a governance-by-design implementation framework addressing key requirements for real-world deployment, including metadata traceability, interoperability based on HL7/FHIR concepts, and documentation elements supporting future regulatory compliance under the European Medical Device Regulation (MDR).
5. Fifth, the feasibility of the proposed framework was explored through a mixed-method evaluation involving community nurses. Technical experiments on synthetic scenarios demonstrated segmentation performance between IoU 0.87 and 0.93 and simulated measurement reliability with ICC values ≥ 0.90, while usability assessment using the SUS indicated good user acceptance (SUS 74.5 ± 6.2).

Finally, the remainder of this paper is organized as follows. Section 2 describes the materials and methods, including the study design, the NURSE-AI framework architecture, the synthetic dataset generation process, and the evaluation methodology. Section 3 presents the results of the technical, interpretability, and usability analyses. Section 4 discusses the main findings, their implications for community healthcare, and the technological and regulatory challenges associated with real-world implementation. Finally, Section 5 provides the conclusions and outlines directions for future research.

## 2. Materials and Methods

### 2.1. Study Design

This study was designed as a pre-implementation feasibility study using a mixed-methods approach to evaluate the methodological, technical, and workflow feasibility of the proposed NURSE-AI framework for multi-sensor wound assessment in community healthcare. The aim was to assess the system before clinical deployment and prior to the use of real patient data.

Accordingly, two complementary components were integrated: (i) a technical evaluation, focusing on segmentation performance and measurement reliability using synthetic multimodal wound scenarios; and (ii) a workflow and usability evaluation, involving FCNs interacting with a prototype interface designed to simulate real-world system use.

This pre-implementation design enabled controlled testing of acquisition workflow, AI outputs, and interpretability mechanisms while avoiding privacy risks and ethical concerns related to early-stage clinical experimentation.

### 2.2. Setting and Participants

The evaluation was conducted within the community nursing services of AUSL Toscana Centro (Italy). Participants consisted of ten FCNs actively involved in home-care services and routine wound assessment activities.

The inclusion criteria were: (i) active professional role within community nursing services; (ii) experience in wound documentation or chronic wound management; and (iii) voluntary participation in the evaluation session.

No exclusion criteria based on seniority were applied, in order to capture heterogeneity in professional experience and routine practice patterns.

### 2.3. Workflow and Experimental Tasks

The evaluation session simulated the workflow of a multi-sensor wound assessment system designed for community care. Participants interacted with a prototype interface that presented synthetic multimodal wound scenarios and guided them through a structured workflow consisting of three sequential phases.

#### 2.3.1. Acquisition Workflow Review

Participants examined the acquisition guidance module, which provided step-by-step prompts designed for home-care environments. These prompts included recommendations regarding camera positioning, stabilization, capture distance, and lighting verification.

#### 2.3.2. Scenario-Based Multimodal Review

Participants reviewed synthetic wound cases presented as aligned multimodal layers, including RGB images, depth maps, thermal patterns, and hyperspectral-proxy perfusion maps.

#### 2.3.3. AI Output Interpretation

Participants evaluated AI-generated wound segmentation outputs and system summaries mapped to WBP domains. During the session, participants were invited to comment on the clarity of acquisition guidance, the interpretability of AI outputs, and the overall usability of the interface.

### 2.4. NURSE-AI Framework

NURSE-AI was developed as a deployment-oriented methodological framework intended to support the evaluation and preparation of multi-sensor wound assessment systems for community healthcare environments.

**Figure 1:**
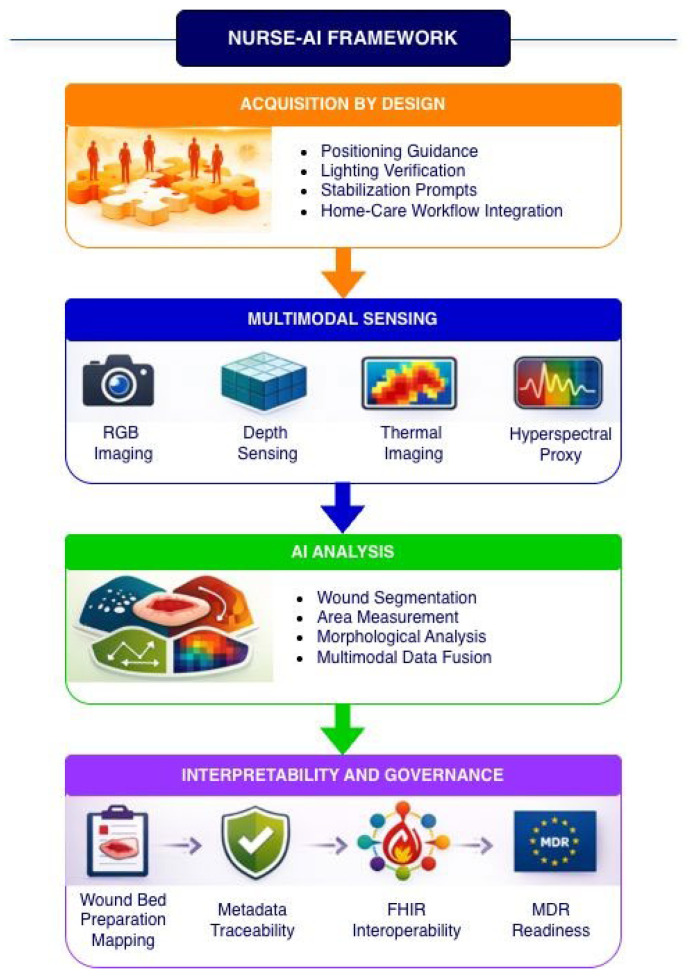
Overview of the NURSE-AI framework. The framework integrates four main modules: (i) acquisition-by-design guidance tailored to community healthcare environments; (ii) generation of a synthetic multimodal dataset including RGB, depth, thermal, and hyperspectral-proxy layers; (iii) AI-based analytics for wound segmentation and extraction of measurement features; and (iv) an interpretability and governance layer aligning AI outputs with Wound Bed Preparation domains while supporting metadata traceability, interoperability, and regulatory readiness.

The framework integrates four functional modules, as illustrated in Figure 1.

#### 2.4.1. Acquisition-by-Design Module

This module defines structured capture guidance tailored to community settings, including positioning prompts, stabilization strategies, and minimal calibration steps suitable for home-care environments.

#### 2.4.2. Synthetic Multimodal Dataset Module

This module generates spatially aligned RGB, depth, thermal, and hyperspectral-proxy layers representing simulated wound scenarios for algorithm testing.

#### 2.4.3. AI Analytics Module

This module processes multimodal data in order to perform wound segmentation and extract geometric features such as wound area and depth estimates.

#### 2.4.4. Interpretability and Governance Module

This module maps AI outputs to clinically interpretable domains derived from the Wound Bed Preparation model and defines metadata requirements supporting traceability, interoperability, and regulatory readiness.

### 2.5. Synthetic Dataset Generation

To enable early-stage testing without the use of patient data, a synthetic multimodal dataset was generated. Each synthetic scenario included four spatially aligned data layers: (i) an RGB layer representing the visual wound surface; (ii) a depth layer approximating wound morphology; (iii) a thermal layer representing simulated inflammatory or ischemic heat patterns; and (iv) a hyperspectral-proxy perfusion layer simulating tissue oxygenation distribution. Synthetic wound morphologies were generated using parametric models designed to reproduce common geometric characteristics observed in chronic wounds, including irregular boundaries, heterogeneous tissue composition, and variable wound depth. Environmental perturbations were introduced in order to simulate real-world acquisition variability in community care environments, including controlled variations in lighting intensity, camera distance, and acquisition angle. Ground-truth annotations were generated for each scenario and included wound segmentation masks and WBP domain labels. Face validity of the generated scenarios was assessed through expert review by clinicians experienced in wound management. However, despite these efforts, synthetic data cannot fully reproduce the biological variability and complexity of real chronic wounds.

### 2.6. AI Analytics Pipeline

AI-based wound segmentation was evaluated using a supervised deep-learning segmentation pipeline applied to the synthetic dataset, following approaches commonly used in automated wound analysis systems [3,4]. The analytical pipeline consisted of the following steps: (i) multimodal data preprocessing and normalization; (ii) automated wound segmentation using a convolutional neural network architecture; and (iii) extraction of wound measurement features, including surface area and depth proxies. The adopted pipeline is consistent with widely used deep learning frameworks for biomedical image segmentation, including convolutional neural networks and encoder–decoder architectures such as U-Net, which have demonstrated strong performance in medical imaging tasks [8,9,10,11]. These architectures enable robust feature extraction and accurate delineation of complex structures, such as irregular wound boundaries, and are widely used as methodological references in medical image analysis [9,10,11].

In addition, recent advances in deep learning-based feature extraction and pattern recognition further support the adopted approach. In particular, temporal–spatial learning methods have demonstrated improved performance and generalization in biomedical data analysis [15], while information-theoretic feature optimization strategies have shown increased robustness in complex data environments [13]. The dataset was divided into training and evaluation subsets to allow controlled performance assessment, following standard practices in machine learning pipelines, where data partitioning is essential to ensure generalization and avoid overfitting. Similar approaches are widely adopted across recent deep learning applications in biomedical data analysis [7,8,9,10,11,12,13,14,15].

Segmentation performance was evaluated using standard computer-vision metrics, including IoU and Dice coefficient. Measurement reliability was evaluated using simulated repeated acquisitions generated through environmental perturbations.

### 2.7. Outcomes and Evaluation Metrics

The study evaluated outcomes across three primary domains:

1. Technical performance. Segmentation accuracy was quantified using IoU, defined as the ratio between the overlap area and the union area of predicted and ground-truth segmentation masks. Measurement repeatability of wound area and depth proxies was assessed using ICC, which quantifies the consistency of repeated measurements under varying acquisition conditions.
2. Interpretability. Agreement between AI-generated WBP domain mapping and nurse interpretation was evaluated using Cohen’s kappa (κ), which measures the level of agreement beyond chance between two classification processes.
3. Usability. Usability of the prototype interface was assessed using the SUS questionnaire administered immediately after the evaluation session.

### 2.8. Statistical Analysis

Descriptive statistics were calculated for all quantitative outcomes. Segmentation performance was summarized using mean IoU values with standard deviation and 95% confidence intervals. Measurement reliability was evaluated using the ICC based on a two-way random-effects model with absolute agreement [ICC (2,1)]. Interpretability agreement between AI outputs and nurse assessment was measured using Cohen’s kappa (κ). Ninety-five percent confidence intervals were calculated for all reliability and agreement statistics. Statistical analyses were performed in Python 3 (Python Software Foundation, https://www.python.org), using the pingouin library for the calculation of intraclass correlation coefficients and Cohen’s kappa, and the NumPy and SciPy libraries for descriptive statistics and confidence intervals. Given the feasibility nature of the study and the limited number of participants involved in usability testing, all analyses were considered exploratory rather than confirmatory.

## 3. Results

### 3.1. Synthetic Segmentation Performance

The segmentation pipeline was evaluated using a synthetic multimodal dataset consisting of four representative wound scenarios. The limited number of scenarios reflects the exploratory nature of this study, which was designed to test the feasibility of the proposed NURSE-AI framework during a pre-implementation phase rather than to provide full algorithm validation. Although limited in number, these scenarios were designed to represent heterogeneous wound characteristics for exploratory testing.

Across the four scenarios, the segmentation algorithm demonstrated stable agreement between predicted and ground-truth wound masks. The mean IoU across scenarios was 0.90, with individual values ranging from 0.87 to 0.93. The corresponding Dice coefficient ranged from 0.93 to 0.96, indicating high segmentation overlap. These values represent segmentation accuracy, indicating the degree of spatial overlap between predicted wound masks and ground-truth annotations.

The segmentation results for each synthetic scenario are reported in Table 1:

### 3.2. Measurement Reliability

Measurement stability was evaluated using simulated repeated acquisitions for each synthetic scenario. Controlled perturbations were introduced to replicate typical variability encountered in community-care image acquisition, including small variations in camera distance, acquisition angle, and lighting conditions.

Wound area estimation suggested high repeatability under simulated conditions, with reliability coefficients indicating stable measurement outputs. The ICC values reflect the repeatability and stability of wound area estimation under simulated acquisition variability.

The measurement reliability results are summarized in Table 2:

### 3.3. Wound Bed Preparation Interpretability Agreement

Agreement between AI-generated WBP domain mappings and nurse interpretation was evaluated using Cohen’s kappa (κ). These values indicate the level of agreement between AI-generated WBP domain classification and nurse clinical interpretation, reflecting the interpretability of the system. Across the evaluated scenarios, agreement levels ranged from κ = 0.80 to κ = 0.84, corresponding to substantial to almost perfect agreement according to the Landis and Koch classification. These findings suggest that the interpretability layer successfully translates multimodal sensor signals and AI outputs into clinically meaningful wound-care domains [14].

Detailed agreement values are reported in Table 3:

### 3.4. Usability Assessment

Usability evaluation was conducted with ten FCNs who interacted with the prototype interface during simulated evaluation sessions. The mean SUS score was 74.5, corresponding to good usability according to standard SUS interpretation thresholds. Individual scores ranged from 65 to 84, indicating generally positive acceptance of the proposed workflow.

Descriptive statistics of the usability assessment are reported in Table 4:

### 3.5. Relationship Between Interpretability and Usability

A moderate positive correlation was observed between WBP-domain interpretability concordance and SUS scores, with a Pearson’s correlation coefficient of r = 0.61. This finding suggests that improved interpretability of AI outputs may positively influence user acceptance of the system.

### 3.6. Robustness Under Acquisition Variability

Segmentation robustness was evaluated under simulated environmental perturbations representing typical variability in community-care acquisition conditions. The reported perturbation values—lighting variation (±15%), distance variation (±10%), and camera tilt (±8°)—represent controlled variation ranges applied around a baseline acquisition condition, rather than discrete experimental settings. Specifically, these values define upper and lower bounds of perturbations introduced during the simulation process to reproduce realistic acquisition variability in home-care environments. Compared with baseline performance (IoU = 0.90), segmentation accuracy showed limited variation: lighting variation (±15%) produced an IoU of 0.88, distance variation (±10%) produced an IoU of 0.89, and camera tilt (±8°) produced an IoU of 0.87. Overall, performance variations remained below 3–4%, suggesting moderate robustness of the segmentation pipeline under realistic acquisition variability. In addition to the evaluated perturbations, other sources of variability may affect real-world performance, including background complexity, partial occlusion, image blur, scale variation, and sensor noise. Although these conditions were not explicitly simulated in the present feasibility study, they represent important directions for future validation in order to provide a more comprehensive assessment of model robustness under realistic community-care acquisition conditions.

The results are summarized in Table 5:

## 4. Discussion

### 4.1. Interpretation of the Main Findings

The results of this feasibility study provide preliminary indications supporting the methodological feasibility of the NURSE-AI framework as a deployment-oriented architecture for AI-enabled wound assessment in community healthcare. These findings should be interpreted within the constraints of a synthetic and preclinical evaluation setting. First, the segmentation results obtained on the synthetic multimodal dataset showed stable agreement between predicted and ground-truth wound masks, with IoU values ranging from 0.87 to 0.93 and Dice coefficients ranging from 0.93 to 0.96. Although these findings were obtained using synthetic scenarios rather than clinical datasets, they indicate that the proposed multimodal processing pipeline can achieve robust segmentation performance under controlled testing conditions. Second, measurement reliability analysis demonstrated excellent repeatability for wound area estimation (ICC = 0.92) and good reliability for depth consistency and volume proxies. These findings are particularly relevant in community healthcare, where acquisition variability during home visits may affect measurement stability. In this regard, the perturbation analysis further showed that segmentation accuracy remained relatively stable under moderate variations in lighting, distance, and camera orientation, thereby supporting the feasibility of the proposed workflow in realistic field conditions. Third, the interpretability layer aligned with the Wound Bed Preparation model demonstrated substantial to almost perfect agreement with nurse assessment, with Cohen’s κ values ranging from 0.80 to 0.84. This suggests that mapping AI outputs onto clinically established wound-care domains may improve the transparency, relevance, and usability of algorithmic outputs for nurses [1,14]. Finally, the usability findings indicated good overall acceptance among FCNs, with a mean SUS score of 74.5. This result suggests that the integration of structured acquisition guidance and clinically interpretable outputs may help support the practical usability of AI-assisted wound assessment systems during routine home-care visits. Taken together, these metrics respectively capture segmentation accuracy (IoU), measurement reliability (ICC), and interpretability alignment with clinical reasoning (Cohen’s κ), thereby supporting the multidimensional feasibility of the proposed framework.

### 4.2. General Considerations

Existing research in wound imaging and sensor-based wound assessment has largely focused on improving algorithmic performance, including segmentation accuracy, geometric reconstruction, and automated measurement of wound area or volume [3,4,5]. While these technical advances are important, many proposed systems have been developed and validated in controlled laboratory environments, where acquisition conditions can be tightly regulated. By contrast, wound care in community healthcare settings takes place in a substantially different operational context. Home environments introduce variability in lighting, patient positioning, available space, and acquisition stability. These factors may significantly influence image quality and measurement reliability, thereby limiting the practical applicability of laboratory-oriented sensor systems. The NURSE-AI framework was therefore designed to address implementation constraints from the outset, prioritizing workflow compatibility and clinical interpretability as primary design principles. Rather than focusing exclusively on algorithmic optimization, the framework emphasizes the integration of sensing technologies into existing nursing workflows, particularly those of Family and Community Nurses involved in chronic wound management. A key component of this approach is the incorporation of an interpretability layer aligned with the Wound Bed Preparation model. By translating AI-generated outputs into clinically meaningful domains, such as tissue status, infection or inflammation signals, moisture balance, and wound edge progression, the system is intended to support clinical reasoning rather than merely provide numerical measurements [1,14].

In this perspective, the present study does not primarily aim to propose a new segmentation algorithm but rather to demonstrate the feasibility of a deployment-oriented methodological architecture for evaluating and preparing AI-enabled wound assessment systems for community healthcare environments.

### 4.3. Translational Impact for Community Healthcare

One of the major challenges in medical AI is the translation of experimental systems into real-world clinical practice. Many AI-enabled imaging solutions demonstrate promising results in controlled settings but face difficulties during implementation because of workflow misalignment, interpretability limitations, or governance and regulatory constraints [14,16]. The NURSE-AI framework addresses this translational gap by integrating four key dimensions within a single methodological architecture: (i) workflow integration, ensuring that acquisition procedures remain feasible in home-care environments; (ii) clinical interpretability, aligning AI outputs with established wound-care reasoning models; (iii) multimodal sensing readiness, enabling the integration of complementary imaging modalities; and (iv) governance-by-design principles, including interoperability, traceability, and regulatory preparedness. This integrated approach provides a structured pathway for developing and evaluating wound imaging systems that are not only technically effective but also operationally compatible with community healthcare services. In particular, the inclusion of workflow-oriented acquisition guidance and clinically grounded interpretability mechanisms may contribute to greater acceptance of AI-assisted wound assessment among community nurses. The usability results observed in this study support the view that these integration strategies can improve user confidence in AI-generated outputs. From a broader perspective, the proposed framework illustrates how sensor-based medical technologies may move beyond purely technical innovation toward implementation-ready healthcare solutions.

Recent research has highlighted the importance of multi-sensor data integration and privacy-aware architectures in healthcare AI systems, which are critical for robust deployment in real-world environments [17,18]. In addition, studies on AI-enabled healthcare services emphasize the role of usability and user acceptance in successful implementation [16]. Furthermore, privacy-preserving approaches such as federated learning are increasingly recognized as key enablers for scalable and secure healthcare AI systems [19].

### 4.4. Technological Challenges

Despite the promising results observed in this feasibility study, several technological challenges remain before multimodal wound imaging systems can be deployed in community healthcare environments. First, robust multimodal data fusion remains a complex technical problem. Combining RGB imaging, depth estimation, thermal sensing, and hyperspectral information requires accurate spatial and semantic alignment while maintaining computational efficiency compatible with mobile or portable devices. Second, acquisition variability in home-care environments may affect sensor reliability. Ambient lighting, camera orientation, patient movement, and limited operator stabilization can introduce noise and reduce measurement precision. Although some of these perturbations were simulated in the present study, future validation in real-world conditions will be necessary. Third, computational constraints must be considered when implementing AI-based wound assessment on mobile platforms. Efficient algorithms capable of real-time or near-real-time processing will be required to ensure practical usability during routine clinical visits. More broadly, these challenges highlight the need for healthcare-oriented multimodal systems that combine methodological robustness with scalability, interoperability, and practical usability in decentralized care environments.

### 4.5. Ethical and Regulatory Considerations

The integration of AI and multimodal sensing technologies in healthcare raises important ethical and governance considerations. One key issue concerns the protection of patient privacy and sensitive health data. To address this challenge during the early development phase, the present study relied on a synthetic multimodal dataset, thereby avoiding the use of real patient data while still enabling controlled evaluation of the proposed framework. This strategy supports GDPR compliance and reduces ethical risks associated with early-stage experimentation involving clinical material. Another important aspect concerns the transparency and interpretability of AI-generated outputs. Systems that provide automated clinical assessments should enable healthcare professionals to understand and contextualize the basis of algorithmic outputs. The WBP-aligned interpretability layer proposed in this study was intended to address this issue by mapping AI-derived signals onto an established wound-care framework [1,14]. Finally, AI-enabled wound assessment systems may potentially fall within the category of Software as a Medical Device (SaMD), thus requiring careful consideration of regulatory requirements, including traceability, validation procedures, and risk management documentation under the European Medical Device Regulation (MDR). Embedding these governance considerations into the design phase, as proposed in the NURSE-AI framework, may facilitate future regulatory approval and responsible clinical adoption.

### 4.6. Limitations

This study has several limitations that should be acknowledged. First, the technical evaluation was based on a synthetic dataset and therefore does not provide definitive evidence of performance in real clinical settings. Synthetic scenarios are useful for early-stage controlled testing, but they cannot fully reproduce the biological and contextual complexity of real chronic wounds. Second, the sample of participating nurses was limited in size and drawn from a single community healthcare setting. As a consequence, usability findings should be interpreted as preliminary. Third, the analyses performed in this study were exploratory rather than confirmatory. The reported quantitative metrics are intended to support feasibility assessment and framework refinement rather than to establish definitive clinical efficacy. These limitations are consistent with the pre-implementation nature of the study and highlight the need for future validation using real patient datasets and multicenter clinical evaluation. Furthermore, the use of a limited number of synthetic scenarios may restrict the generalizability of the reported performance metrics. While a broad range of artificial intelligence applications exists across different domains, this study deliberately prioritizes the domain-specific literature directly relevant to biomedical imaging and wound assessment in order to maintain conceptual coherence. This selective approach avoids the inclusion of references that are not directly translatable to the clinical and technological context of the proposed framework, while still incorporating recent methodological contributions that strengthen the scientific background of the study [7,8,9,10,11].

## 5. Conclusions

This study presented NURSE-AI, a Nurse-by-Design methodological framework for the evaluation and preparation of multimodal sensor-based wound assessment systems in community healthcare. Through a pre-implementation feasibility evaluation involving community nurses and synthetic multimodal scenarios, the study demonstrated the potential value of combining workflow-oriented system design with interpretable AI outputs. In particular, synthetic multimodal testing showed segmentation performance with IoU values ranging from 0.87 to 0.93, measurement reliability with ICC values ≥ 0.90 for wound area estimation, and interpretability agreement between AI outputs and nurse assessment with Cohen’s κ ranging from 0.80 to 0.84. In addition, usability evaluation indicated good user acceptance, with a System Usability Scale (SUS) score of 74.5 ± 6.2. These findings suggest that integrating structured acquisition guidance, multimodal visualization, and clinically meaningful interpretability may support the usability and acceptance of AI-assisted wound assessment systems in community care. Future research should focus on clinical validation using real patient datasets, the development of real-time multimodal data fusion pipelines, multicenter evaluation of nursing workflows in community healthcare environments, and integration with electronic health record systems using FHIR-based interoperability interfaces. By supporting the transition from experimental sensing technologies toward implementation-ready healthcare systems, the NURSE-AI framework may contribute to supporting future improvements in the accuracy, consistency, and interpretability of chronic wound assessment in community-based care.

## Figures and Tables

**Table 1:** Segmentation performance across synthetic wound scenarios: segmentation accuracy evaluated across four synthetic multimodal wound scenarios. Intersection over Union (IoU) and Dice coefficient values represent overlap between predicted segmentation masks and ground-truth annotations.

Scenario	IoU	Dice
Scenario 1	0.87	0.93
Scenario 2	0.89	0.94
Scenario 3	0.91	0.95
Scenario 4	0.93	0.96

**Table 2:** Measurement reliability across synthetic scenarios: reliability analysis of wound measurement parameters under simulated repeated acquisitions. Reliability was evaluated using the Intraclass Correlation Coefficient (ICC), with interpretation based on commonly accepted reliability thresholds.

Measurement Parameter	Reliability (ICC)	Interpretation
Wound area	0.92	Excellent
Depth consistency	0.86	Good
Volume proxy stability	0.85	Good

**Table 3:** Interpretability agreement between AI outputs and nurse assessment. Agreement between AI-generated Wound Bed Preparation domain classification and nurse interpretation measured using Cohen’s kappa statistic.

WBP Domain	Cohen’s κ	Interpretation
Tissue status	0.84	Almost perfect
Infection/inflammation	0.81	Substantial
Moisture balance	0.80	Substantial
Wound edge progression	0.82	Substantial

**Table 4:** SUS results: descriptive statistics of SUS scores obtained from participating community nurses after interacting with the NURSE-AI prototype interface.

Statistic	Value
Mean SUS score	74.5
Standard deviation	6.2
Minimum	65
Maximum	84

**Table 5:** Segmentation robustness under simulated perturbations: segmentation accuracy evaluated under simulated acquisition perturbations representing typical variability in community healthcare environments.

Perturbation Type	IoU
Baseline	0.90
Lighting variation ±15%	0.88
Distance variation ±10%	0.89
Camera tilt ±8°	0.87

## Data Availability

The data presented in this study are based on synthetic multimodal scenarios generated for methodological feasibility assessment. Further details are available from the corresponding author upon reasonable request.
